# Development of a New LAMP Assay for the Detection of *Ancylostoma caninum* DNA (Copro-LAMPAc) in Dog Fecal Samples

**DOI:** 10.3389/fvets.2021.770508

**Published:** 2021-11-12

**Authors:** Héctor Gabriel Avila, Marikena Guadalupe Risso, Marta Cabrera, Paula Ruybal, Silvia Analía Repetto, Marcos Javier Butti, Marcos David Trangoni, Graciela Santillán, Verónica Mirtha Pérez, María Victoria Periago

**Affiliations:** ^1^Laboratorio Provincial de Zoonosis de San Juan, Facultad de Ciencias Veterinarias, Facultad de Ciencias Químicas y Tecnológicas, Universidad Católica de Cuyo, San Juan, Argentina; ^2^Consejo Nacional de Investigaciones Científicas y Técnicas, Buenos Aires, Argentina; ^3^Departamento de Microbiología, Facultad de Medicina, Universidad de Buenos Aires, Buenos Aires, Argentina; ^4^Instituto de Investigaciones en Microbiología y Parasitología Médica, Consejo Nacional de Investigaciones Científicas y Técnicas (CONICET) - Universidad de Buenos Aires, Buenos Aires, Argentina; ^5^Departamento de Parasitología, Instituto Nacional de Enfermedades Infecciosas, Administración Nacional de Laboratorios e Institutos de Salud (ANLIS) “Dr. Carlos G. Malbrán”, Buenos Aires, Argentina; ^6^División Infectología, Hospital de Clínicas “José de San Martín”, Universidad de Buenos Aires, Buenos Aires, Argentina; ^7^Laboratorio de Parasitosis Humanas y Zoonosis Parasitarias, Cátedra de Parasitología Comparada, Facultad de Ciencias Veterinarias, Universidad Nacional de La Plata, La Plata, Argentina; ^8^Laboratorio de Brucella, Campylobacter y Microbiota del rumen, Instituto de Biotecnología/Instituto de Agrobiotecnología y Biología Molecular, Unidades Ejecutoras de Doble Dependencia (UEDD) INTA-CONICET, Investigación en Ciencias Veterinarias y Agronómicas (CICVyA), Centro Nacional de Investigaciones Agropecuarias (CNIA), INTA Castelar, Buenos Aires, Argentina; ^9^Sección de Rabia y Zoonosis, Dirección de Epidemiología, Ministerio de Salud Pública de San Juan, San Juan, Argentina; ^10^Fundación Mundo Sano, Buenos Aires, Argentina

**Keywords:** loop mediated isothermal amplification, *Ancylostoma caninum*, copro-diagnosis, ancylostomiasis, molecular diagnosis

## Abstract

*Ancylostoma caninum* is a zoonotic nematode which is able to affect animals and humans. Diagnosis in the definitive host and environmental detection are key to prevent its dissemination and achieve control. Herein, a new coprological LAMP method for the detection of *A. caninum* (Copro-LAMPAc) DNA was developed. DNA extraction was performed using a low-cost method and a fragment of the *cox*-1 gene was used for primer design. The analytical sensitivity, evaluated with serial dilutions of genomic DNA from *A. caninum* adult worms, was 100 fg. A specificity of 100% was obtained using genomic DNA from the host and other pathogens. The Copro-LAMPAc was evaluated using environmental canine fecal samples. When compared with gold standard optical microscopy in epidemiological studies, it proved to be more sensitive. This new LAMP assay can provide an alternative protocol for screening and identification of *A. caninum* for epidemiological studies in endemic areas.

## Introduction

Over one-third of the worldwide population harbors a parasitic helminth; these parasitic diseases generate millions of deaths each year (1) and produce a series of morbidities that affect mostly vulnerable populations from low and medium income countries (LMIC), causing millions of disability-adjusted life years (DALYs) (2). *Ancylostoma caninum* is a zoonotic nematode, proven to cause local lesions (e.g., papular/pustular eruptions) rather than serpiginous tracks typical of cutaneous larva migrans. Other conditions occasionally attributed to *A. caninum* are myositis, unilateral subacute neuroretinitis, and eosinophilic enteritis (3). The filariform larvae stage penetrates the human epidermis but typically does not develop in the intestine, therefore, it becomes trapped in the skin and underlying muscles, causing irritation and itching (4–6). In some studies, few cases were reported where *A. caninum* was able to complete its migration to the human intestine (7), generating several eosinophilic gastroenteritis (8). Recent studies suggests that patent human infection is a possibility (9, 10).

Infection in dogs can occur through percutaneous penetration of third-stage larvae, orally or through lactogenic and trans-mammary route (11). Previously infected rodents and insects can act as paratenic and transport hosts, respectively. Canids ingesting such a host will develop patent infections due to reactivation of hypobiotic larvae in the prey (12). In puppies, symptoms can be extensive, including, anemia diarrhea, malnutrition and death. In older dogs, symptoms are mostly limited to anemia (13). Immature worms may still produce clinical disease (i.e., no eggs observed in feces). In Argentina, previous epidemiological studies showed a variable range of *A. caninum* prevalence in canine feces (14–26). A study performed in the central Buenos Aires province showed that 60.5% of dogs were parasitized with *A. caninum* (27). In contrast to the southern Patagonian region of the country, where the reported prevalence of *A. caninum* in canines was lower (0.41–6.2%) (14, 22).

The detection of parasitic structures in the feces is used for detection of infection, using different coproparasitological techniques to concentrate the sample and increase sensitivity (13). The most common coproparasitological techniques used for the detection of *A. caninum* eggs in canine feces is the standard fecal flotation technique with either saturated salt (28) or sucrose (29). The main disadvantages of these techniques, in epidemiological studies, is associated to false negative results due to low parasite burden, the biology of the parasite, and human resources that are not specifically trained or have little experience in the identification of parasitic structures under the microscope (30). Nonetheless, there are alternative coproparasitological methods that may be used to improve the sensitivity (31). Copro-antigen detection methods through Enzyme-Linked Immuno Sorbent Assay (ELISA) have shown a wide range of sensitivity and specificity values (32). Moreover, the detection of nucleic acids through the use of molecular techniques has generated improvements in sensitivity and specificity values (33–36). However, these techniques cannot generally be performed in endemic areas because they require sophisticated equipment and highly qualified personnel, complicating its implementation.

On the other hand, the isothermal amplification of nucleic acids has begun to be widely used since it can be carried out in laboratories without specialized equipment. Particularly, the LAMP (loop-mediated isothermal amplification) technique (37), uses 3 primer pairs that recognize a small DNA fragment, and generate looped structures that serve as a template to start a new polymerization cycle thus providing both higher specificity and sensitivity (38). The DNA polymerase I from *Bacillus stearothermophilus* (*Bst*) used in the technique causes DNA strand displacement and therefore does not require denaturing the double strand, thus the technique can be performed with any equipment that guarantees a constant temperature (37). The LAMP reaction characteristics (affordable, sensitive, specific, user-friendly, rapid, equipment-free) make it an attractive method for use in diagnosis and epidemiological surveillance (39).

Different protocols based on LAMP reactions, have been implemented for diagnosis of different parasites (30, 38, 40–49). Similarly, LAMP reactions have been shown to be useful for the detection of pathogens in food and surveillance of water quality (50–57). Nonetheless, this technique is not currently widely used in the diagnostic routine, probably due to the high cost of the visualization methods required and the cost of DNA extraction. Another complication is the contamination of the sample with unwanted amplification products which can be avoided by correcting the workflow.

In this work the development of an easy copro-LAMP reaction for *A. caninum* detection was performed. Furthermore, this reaction was evaluated using two different methods for DNA extraction in order to be able to implement it in laboratories without sophisticated equipment.

## Materials and Methods

### Samples and Parasite Isolation

All canine fecal samples collected in San Juan and Corrientes Province, Argentina, were stored at −80°C for a week to ensure inactivation. *A. caninum* adult worms were kindly provided by Dr. Marcos Butti (National University of La Plata, Buenos Aires province, Argentina) and were preserved in 70% ethanol.

### Optical Microscopy

Three standard concentration methods were employed for detection of hookworm eggs: two different flotation techniques, one with sugar and one with salt (28, 29), and the Telemann sedimentation technique (58). The techniques chosen for this study are standard concentration techniques that increase the chances of detecting parasitic structures, including nematode parasites such as *Ancylostoma* sp. Each sample was microscopically examined at 100 X and 400 X magnifications.

### DNA Extraction

DNA extraction from host, bacteria and parasites was performed using different protocols. The adult nematode DNA was obtained using an already published protocol (59). The DNA from adult cestode parasites was obtained using the DNeasy Blood & Tissue Kit^®^ (QIAGEN, Germantown, MD, USA). While DNA from *Escherichia coli* was extracted using the phenol-chloroform method (60). Finally, DNA from the intestinal tissue of the host was extracted following the manufacturer's instructions for the DNeasy Blood & Tissue Kit^®^ (QIAGEN, Germantown, MD, USA).

The DNA integrity and concentrations were determined according to Avila et al. (38). The DNA extraction and purification from feces (fDNA) was obtained using a commercial kit (CKM) and an alternative low-cost method (LCM) based on mesh-filtration of stool, followed by alkaline hydrolyses, according to Avila et al. (38).

### LAMP Assay

#### Primer Design

The selection of the target gene for the primer design was performed according to Avila et al. (38), using Primer V5 design software (61). The target for primer design was a 208 bp region of the mitochondrial gene *cox*-1 (Genbank accession number NC_012309.1, region: 293-501 bp). This selected region guaranteed the necessary specificity for primer generation, according to previous descriptions (38, 49, 62).

#### Master Mix

The Copro-LAMPAc reaction was performed in a 12.5 μl final reaction mixture containing: 20 mM Tris (pH 8.8), 50 mM KCl, 8 mM MgSO_4_, 10 mM (NH_4_)_2_SO_4_, 8 mM betaine, 1.4 mM dNTPs and 4 U *Bst* 2.0 polymerase (New England Biolabs, Ipswich, MA, United States). The primer concentration was 0.02 nmol of FIP and BIP primer, 0.0025 nmol of F3 and B3 primer, and 0.005 nmol of LB, LF primer and 1 μl of DNA as template or water for negative controls. All reactions were performed on ice. The amplification conditions were evaluated using a temperature gradient (52–62°C) and different incubation times (15–120 min). The results were obtained using 1 μl of 1000X SYBR Green I^®^ (Thermo Fisher Scientific, Waltham, MA, USA).

#### Analytical Sensitivity

Ten-fold ultrapure water serial dilutions of gDNA from *A. caninum* were used to measure the analytical sensitivity for detection.

#### Specificity Evaluation

The specificity of the Copro-LAMPAc was evaluated by using 10 pg of gDNA of canine and bacterial DNA, which are always present. Additionally, the specificity was evaluated with DNA from other helminth parasites which are usually present in canine feces: *Toxocara canis, T. cati, Toxascaris leonina, Echinococcus granulosus sensu stricto, Dipylidium caninum*, and *Taenia hydatigena*. Particularly, the *T. hydatigena* cox-1 region, has high similarity with corresponding regions of other *Taenia* species, e.g., *T. pisiformis* (63).

#### Environmental Samples From Endemic Areas

Forty-one environmental fecal samples were collected from San Juan and Corrientes Provinces, Argentina. Samples were analyzed by optical microscopy and Copro-LAMPAc assay using triplicate DNA samples obtained by both CKM and LCM methods.

## Results

### LAMP Design and Standardization

**Figure 1:**
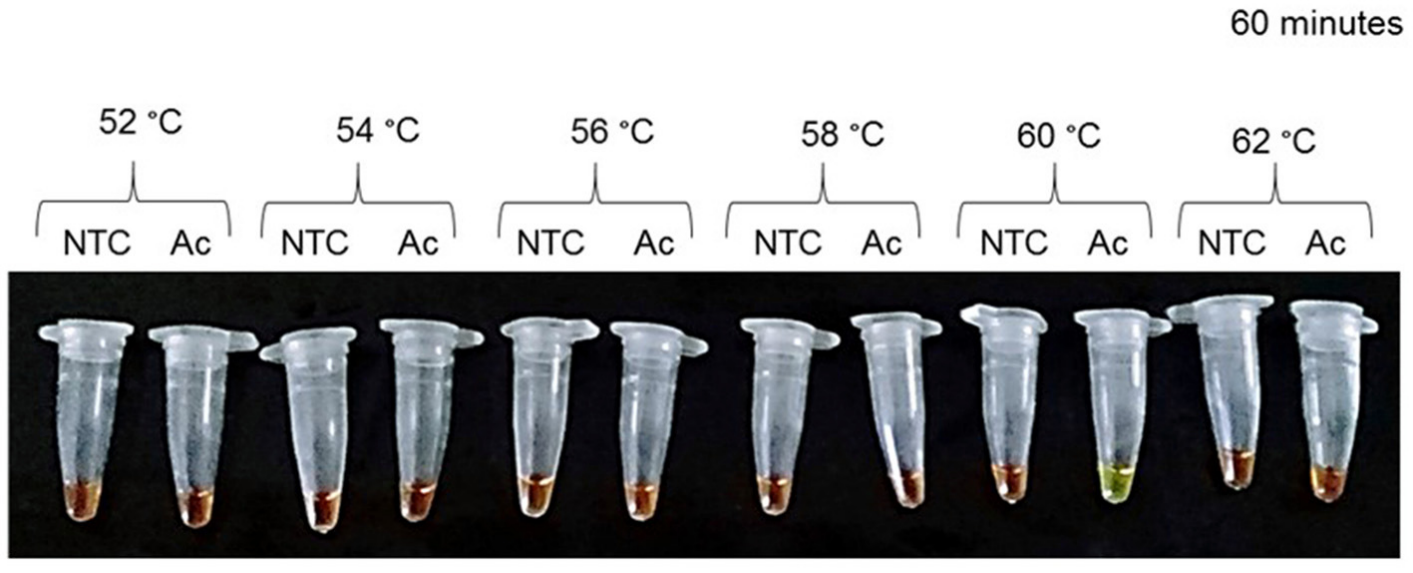
Optimal conditions for *Ancylostoma caninum* genomic DNA amplification. Incubation temperature optimization at a range of 52–62°C. During a 60 min incubation, a temperature of 60°C was considered optimal. Results were visualized using SYBR Green I^®^ 1000 X. NTC, negative control; Ac, *Ancylostoma caninum*.

The primer set was designed using an *A. caninum cox*-1 fragment gene. The primer sets (Table 1) were selected according to Avila et al. (38). Optimal temperature incubation was 60°C for 1 h (Figure 1; Supplementary Table 1), with a final incubation at 80°C for 15 min.

**Table 1:** Copro-LAMPAc primer set: sequence data of the primer set designed for *Ancylostoma caninum* detection.

**Primer**	**Sequence**
FIP-Ac	CTGTTCAACTAGTACCACAACCTATGTT TTTGATTGTTACCTACTGCTA
BIP-Ac	ACCCAGAGTTCTTAAAGGAGGATATT GGCGATTTTTAGTTTACATTG
F3-Ac	CGGATATAAGTTTTCCTCGTTTA
B3-Ac	ACCTAAAATTGAACTCAAACCA
LF-Ac	AGATTCTTGTTTTGTTGAT
LB-Ac	CACCCGGGTAGAAGAGTGG

*This primer set was designed for the specific recognition of a mitochondrial cox1fragment*.

**Figure 2:**
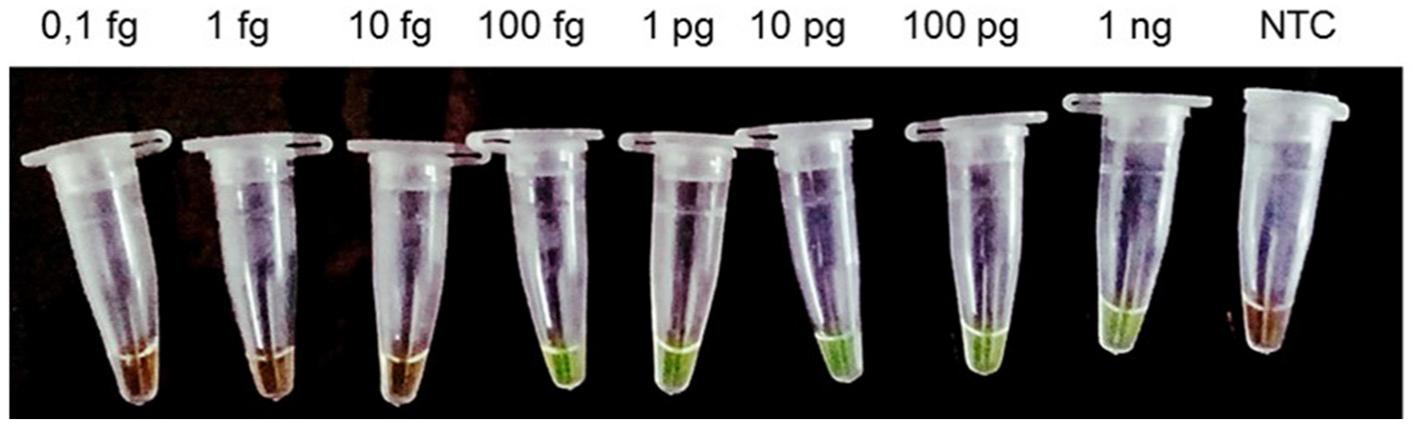
Analytical sensitivity of the Copro-LAMPAc assay. Serial dilutions of *Ancylostoma caninum* genomic DNA (1 fg−1 ng) were used to determine the limit of detection. One microliter of SYBR Green I^®^ 1000 X (Thermo Fisher Scientific, Waltham, MA, United States) was used view the results. NTC, negative control.

**Figure 3:**
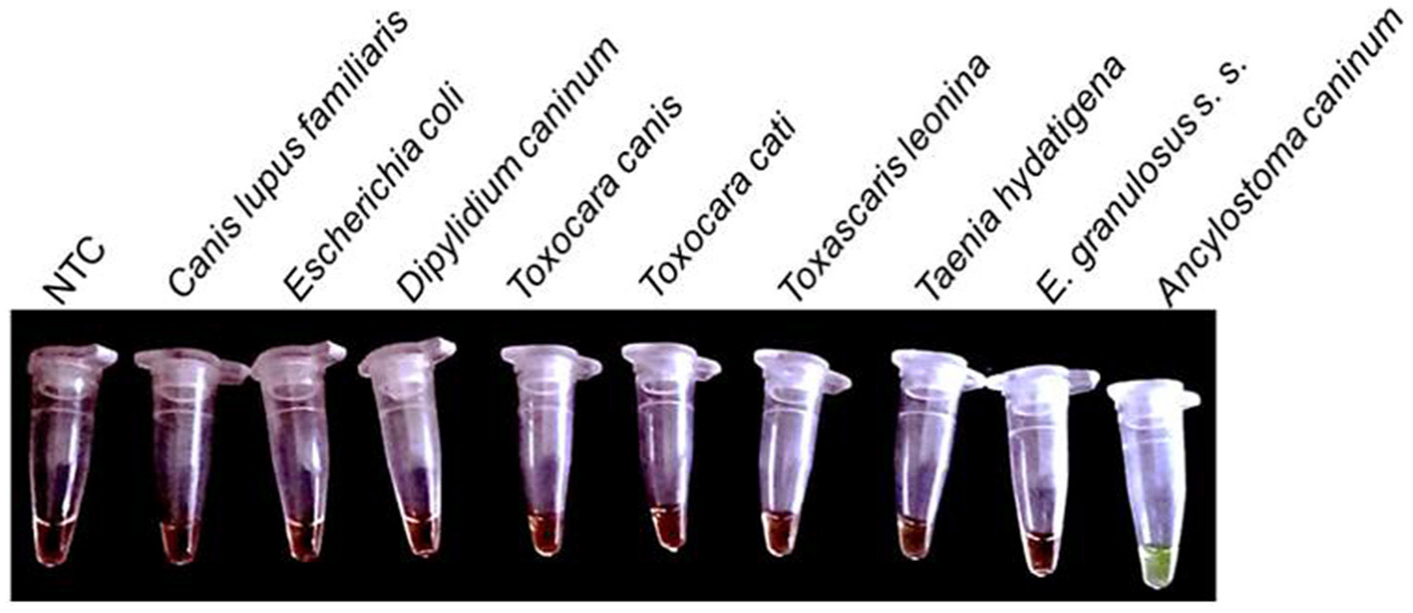
Specificity of the Copro-LAMPAc assay. Cross reaction was not observed when 10 pg of DNA from *Canis lupus familiaris, Escherichia coli, Dipylidium caninum, Taenia hydatigena, Toxascaris leonina, Toxocara canis, Toxocara cati* and *Echinococcus granulosus sensu stricto*, were used. NTC, negative control; *A. caninum*, 1 pg of genomic DNA.

The Copro-LAMPAc reaction was able to detect up to 100 fg of gDNA from *A. caninum* (Figure 2); cross reaction was not observed with either the host, bacterial DNA or DNA from the other parasites tested (Figure 3).

### Environmental Samples Analysis

Forty-one canine fecal samples collected from the environment in Corrientes and San Juan provinces, were analyzed. Using optical microscopy methods, twelve samples (29.3%) were positive for hookworm eggs (Table 2). On the other hand, the Copro-LAMPAc assay was able to detect *A. caninum* DNA from 22 samples (53.7%). This result was not affected by the method of DNA extraction (CKM or LCM); this result was confirmed in triplicate.

**Table 2:** Detection of *Ancylostoma caninum* in environmental samples.

	**Optical microscopy**	**LAMP-Ac +**	**LAMP-Ac +**
		**commercial kit**	**low-cost method**
Positive	12 (29.3%)	22 (53.7%)	22 (53.7%)
Negative	29 (70.7%)	19 (46.3%)	19 (46.3%)
Total	41 (100%)	41 (100%)	41 (100%)

*Each positive result was considered according to the presence of hookworm eggs (optical microscopy) or visual orange-green conversion (LAMP). Identical results were obtained using DNA obtained by either a commercial kit or a low-cost method. Triplicates were used for each result*.

## Discussion

Hookworms are nematodes that can affect human and animals (3, 64, 65). *Ancylostoma* diagnosis is not provided at the species level since the morphology of *Ancylostoma* egg species is often indistinguishable. The simplicity of copromicroscopy and its specificity makes it the gold standard method for the parasitic helminth egg detection. However, this copromicroscopy presents the disadvantage of low sensitivity in epidemiological studies, or in the identification of the hookworm species. These problems in sensitivity and specificity values can be resolved using molecular biology. Unfortunately, the high costs for its realization make it difficult to use in laboratories with poor resources or the lack of equipment specific for PCR. This is the reason why many PCR protocols specific for *A. caninum* detection that have been previously developed (33–35, 66, 67), are not currently available in some endemic areas.

Isothermal amplification techniques, such as LAMP, are able to specifically and sensitively amplify nucleic acids, without the need of expensive equipment. These characteristics make them an attractive alternative for molecular diagnosis in epidemiological studies (30, 68, 69). Different LAMP protocols for helminth DNA detection were developed. The LAMP assay for *E. granulosus* s. s. DNA detection developed by Ni et al. (70), was able to detect 10 pg of gDNA, and was more sensitive than both copro-ELISA and copro-PCR. While Bucher et al. (71) developed an easy and cost-effective protocol for *E. multilocularis* copro-detection, some LAMP reactions for the detection of nematode DNA were not able to overcome the sensitivity values of gold standard techniques (72) or PCR methods (73).

Herein, we developed for the first time, a simple LAMP reaction for *A. caninum* copro-detection. The Copro-LAMPAc was able to detect 100 fg of gDNA from *A. caninum* adult worms, which is more sensitive than previously reported values for other PCR assays (33–35, 66). This analytical sensitivity value is included in the femtograms range, similar to what has been obtained in other studies (38, 49, 74). In future studies, the *A. caninum* egg limit of detection could be determined.

The Copro-LAMPAc assay proved to be more sensitive than optical microscopy for the identification of *A. caninum* (Table 2). As previously described (38), commercial kits for DNA extraction from stool can be replaced by more accessible methods. This significant cost-reduction in sample processing increases the possibility of the Copro-LAMPAc assay implementation in practically any laboratory. It could be compared to techniques that increase the sensitivity of conventional coprological methods, such as the FLOTAC or mini-FLOTAC (31), although these methods cannot be used to distinguish hookworm species.

Due to the zoonotic potential of *A. caninum*, which can cause skin damage, eosinophilic enteritis or patent infection in humans, it is important to be able to have a tool to monitor the environmental contamination by hookworms (and other nematodes, such as Ascarids), since free-roaming domestic animals are a primary source of contamination (75, 76). Additionally, recent studies have demonstrated the presence of drug resistance to anthelminthics in dog hookworms (*A. caninum*) (77–79), where the authors suggest the use of the fecal egg count reduction test to evaluate the efficacy of drugs used (79). In epidemiological studies, the efficacy of this technique is lower. Therefore, we believe that other important uses for the copro-LAMPAc test could be for epidemiological studies, screenings, and when a species-diagnosis for hookworms is needed in the context of a veterinary clinic.

The Copro-LAMPAc assay developed herein is promising in the light of the sensitivity/specificity values herein obtained for *A. caninum* detection. The Copro-LAMPAc protocol may be implemented for epidemiological purposes in low complexity laboratories. Nevertheless, given the results obtained here are preliminary, future wider field studies should be performed to validate these findings. If the larger scale is confirmed, this method could also be implemented to determine environmental contamination. Adaptations to this protocol could be performed in order to detect *A. caninum* contamination in other matrixes (i.e., food, water, sand, etc.), in order to prevent infection in both animals and humans.

## Conclusions

The Copro-LAMPAc technique provides a specific and sensitive method for the *A. caninum* detection, a zoonotic parasite that affects both humans and animals. Moreover, this technique uses an accessible method for DNA extraction, providing an easy and low cost tool for diagnosis. This study provides a new protocol to improve hookworm screening for epidemiological studies in basic laboratories from endemic areas.

## Data Availability Statement

The original contributions presented in the study are included in the article/Supplementary Material, further inquiries can be directed to the corresponding author/s.

## Ethics Statement

The parasite material was obtained from the cadaver of animals previously donated by their respective owners, to the University Hospital from the School of Veterinary of the National University of La Plata.

## Author Contributions

MP and HA conceptualized and designed the experiments. HA performed the experiments and wrote the manuscript. MR, MC, PR, SR, MB, MT, and GS contributed reagents, materials, and/or analytical tools, and wrote the manuscript. MP and VP critically revised and approved the manuscript. All authors contributed to the article and approved the submitted version.

## Funding

This work was supported by Fundación Mundo Sano, Consejo Nacional de Investigaciones Científicas y Tecnológicas (CONICET), Ministerio de Salud Pública de la Provincia de San Juan and Universidad Católica de Cuyo.

## Conflict of Interest

The authors declare that the research was conducted in the absence of any commercial or financial relationships that could be construed as a potential conflict of interest.

## Publisher's Note

All claims expressed in this article are solely those of the authors and do not necessarily represent those of their affiliated organizations, or those of the publisher, the editors and the reviewers. Any product that may be evaluated in this article, or claim that may be made by its manufacturer, is not guaranteed or endorsed by the publisher.

## References

[1] King CH. Health metrics for helminth infections. Acta Trop. (2015) 141:150–60. 10.1016/j.actatropica.2013.12.001 24333545 PMC4055550

[2] Shepherd C Wangchuk P Loukas A. Of dogs and hookworms: man's best friend and his parasites as a model for translational biomedical research. Paras Vectors. (2018) 11:59. 10.1186/s13071-018-2621-2 29370855 PMC5785905

[3] Morelli S Diakou A Di Cesare A Colombo M Traversa D. Canine and feline parasitology: analogies, differences, and relevance for human health. Clin Microbiol Rev. (2021) 34:e0026620. 10.1128/CMR.00266-20 34378954 PMC8404700

[4] Kwon IH Kim HS Lee JH Choi MH Chai JY Nakamura-Uchiyama F . A serologically diagnosed human case of cutaneous larva migrans caused by *Ancylostoma caninum*. Korean J Parasitol. (2003) 41:233–237. 10.3347/kjp.2003.41.4.233 14699264 PMC2717515

[5] Bowman DD Montgomery SP Zajac AM Eberhard ML Kazacos KR. Hookworms of dogs and cats as agents of cutaneous larva migrans. Trends Parasitol. (2010) 26:162–7. 10.1016/j.pt.2010.01.005 20189454

[6] Heukelbach J Feldmeier H. Epidemiological and clinical characteristics of hookworm-related cutaneous larva migrans. Lancet Infect Dis. (2008) 8:302–9. 10.1016/S1473-3099(08)70098-7 18471775

[7] Croese J Loukas A Opdebeeck J Fairley S Prociv P. Human enteric infection with canine hookworms. Ann Intern Med. (1994) 120:369–74. 10.7326/0003-4819-120-5-199403010-00003 8304653

[8] Walker NI Croese J Clouston AD Parry M Loukas A Prociv P. Eosinophilic enteritis in Northeastern Australia: pathology, association with *Ancylostoma caninum*, and implications. Am J Surg Pathol. (1995) 19:328–37. 10.1097/00000478-199503000-00011 7872431

[9] Furtado L Dias L Rodrigues T Silva V Oliveira VN Gomes Mendes de R . Egg genotyping reveals the possibility of patent *Ancylostoma caninum* infection in human intestine. Sci Rep. (2020) 10:3006. 10.1038/s41598-020-59874-8 32080267 PMC7033205

[10] Ngcamphalala PI Lamb J Mukaratirwa S. Molecular identification of hookworm isolates from stray dogs, humans and selected wildlife from South Africa. J Helminthol. (2020) 94:e39. 10.1017/S0022149X19000130 30789121

[11] Dwight DB. Georgis' Parasitology For Veterinarians, 10 th Edition. Vol. 4 (2014). p. 179–83.

[12] Strube C Mehlhorn H. Dog Parasites Endangering Human Health. Vol. 9 (2021). p. 147–90.

[13] Dantas-Torres F Ketzis J Mihalca AD Baneth G Otranto D Tort GP . TroCCAP recommendations for the diagnosis, prevention and treatment of parasitic infections in dogs and cats in the tropics. Vet Parasitol. (2020) 283:109167. 10.1016/j.vetpar.2020.109167 32580071

[14] Sánchez P Raso S Torrecillas C Mellado I Ñancufil A Oyarzo CM . Contaminación biológica con heces caninas y parásitos intestinales en espacios públicos urbanos en dos ciudades de la Provincia del Chubut: Patagonia Argentina. Parasitol Latinoam. (2003) 58:1315. 10.4067/S0717-77122003000300008

[15] Alonso JM López MA Bojanich M V. Infección por Toxocara canis en población adulta sana de un área subtropical de Argentina. Parasitol Latinoam. (2004) 59:61–4. 10.4067/S0717-77122004000100012

[16] Taranto NJ Passamonte L Marinconz R De Marzi MC Cajal SP Malchiodi EL. Parasitosis zoonoticas transmitidas por perros en el Chaco Salteño. Medicina. (2000) 60:217–20. 10962811

[17] Lechner L Denegri G Sardella NH. Evaluación del grado de contaminación parasitaria en plazas de la ciudad de Mar del Plata, Argentina. Rev Vet. (2005) 16:53–6. 10.30972/vet.1914303

[18] Andresiuk V Rodríguez F Denegri G Sardella N Hollmann P. Relevamiento de parásitos zoonóticos en materia fecal canina y su importancia para la salud de los niños. Arch Argent Pediatr. (2004) 102:325–9.

[19] Rodríguez F Denegri G Sardella N Hollmann P. Relevamiento coproparasitológico de caninos ingresados al Centro Municipal de Zoonosis de Mar del Plata, Argentina. Rev Vet. (2005) 16:9. Available online at: https://revistas.unne.edu.ar/index.php/vet/article/view/1984

[20] Passucci J West M. Parasitosis interna en un albergue de perros en la ciudad de Tandil, 1995. Pets. (1996) 12:4.

[21] Milano AMF Oscherov EB. Contaminación de aceras con enteroparásitos caninos en Corrientes, Argentina. Parasitol Latinoam. (2005) 60:82–85. 10.4067/S0717-77122005000100015

[22] Soriano SV Pierangeli NB Roccia I Bergagna HF Lazzarini LE Celescinco A . A wide diversity of zoonotic intestinal parasites infects urban and rural dogs in Neuquén, Patagonia, Argentina. Vet Parasitol. (2010) 167:81–5. 10.1016/j.vetpar.2009.09.048 19864068

[23] Larrieu E Alvarez E Cavagion L Lanbert J Calvo C Herrasti A . Estudio descriptivo de la contaminación por materia fecal de pequeños animales en áreas urbanas de General Pico, Argentina. Vet Arg. (1997) 14:198–200.

[24] Rimoldi P Negro P. Zoonosis parasitarias y educación para la salud. (Thesis) UNR (2007).

[25] Battistoni B Florencia M. Parásitos de importancia zoonótica en perros y gatos con dueños de la localidad de Esperanza (Santa Fe) (thesis). UNL, Santa Fe, Argentina (2015) 9:1–2.

[26] Rivero MR De Angelo C Nuñez P Salas M Motta CE Chiaretta A . Environmental and socio-demographic individual, family and neighborhood factors associated with children intestinal parasitoses at Iguazú, in the subtropical northern border of Argentina. PLoS Negl Trop Dis. (2017) 11:e0006098. 10.1371/journal.pntd.0006098 29155829 PMC5714390

[27] Gamboa MI Corbalán VV Paladini A. Zoonosis parasitarias en caninos de un área vulnerable. Rev Enfermedades Infecciosas Emergentes. (2020) 15:39-44

[28] Willis H. A simple levitation method for the detection of hookworm ova. Med J Aust. (1921) 29:375–6. 10.5694/j.1326-5377.1921.tb60654.x

[29] Sheather L. The detection of intestinal protozoa and mange parasites by a floatation technique. J Pathol Ther. (1923) 36:266–75. 10.1016/S0368-1742(23)80052-2

[30] Deng MH Zhong LY Kamolnetr O Limpanont Y Lv ZY. Detection of helminths by loop-mediated isothermal amplification assay: a review of updated technology and future outlook. Infect Dis Poverty. (2019) 8:20. 10.1186/s40249-019-0530-z 30905322 PMC6432754

[31] Maurelli MP Rinaldi L Alfano S Pepe P Coles GC Cringoli G. Mini-FLOTAC, a new tool for copromicroscopic diagnosis of common intestinal nematodes in dogs. Paras Vect. (2014) 7:356. 10.1186/1756-3305-7-356 25095701 PMC4262189

[32] Elsemore DA Geng J Cote J Hanna R Lucio-Forster A Bowman DD. Enzyme-linked immunosorbent assays for coproantigen detection of *Ancylostoma caninum* and *Toxocara canis* in dogs and *Toxocara cati* in cats. J Vet Diagnostic Investig. (2017) 29:645–53. 10.1177/1040638717706098 28424002

[33] Rehman A Akhtar R Akbar H Riaz F Rashid I Shehzad W . First report of the molecular detection of *Ancylostoma caninum* in Lahore, Pakistan: the threat from pets. Vet Med. (2017) 62:559–64. 10.17221/14/2017-VETMED

[34] Liu Y Zheng G Alsarakibi M Zhang X Hu W Lu P . Molecular identification of ancylostoma caninum isolated from cats in southern china based on complete ITS sequence. Biomed Res Int. (2013) 2013:868050. 10.1155/2013/868050 24175305 PMC3794661

[35] Hu W Wu S Yu X Abullahi AY Song M Tan L . A Multiplex PCR for simultaneous detection of three zoonotic parasites ancylostoma ceylanicum, *A. caninum*, and Giardia lamblia Assemblage A. Biomed Res Int. (2015) 2015:406168. 10.1155/2015/406168 26447336 PMC4568324

[36] Ngui R Lim YAL Chua KH. Rapid detection and identification of human hookworm infections through high resolution melting (HRM) analysis. PLoS ONE. (2012) 7:e41996. 10.1371/journal.pone.0041996 22844538 PMC3406038

[37] Notomi T Okayama H Masubuchi H Yonekawa T Watanabe K Amino N . Loop-mediated isothermal amplification of DNA. Nucleic Acids Res. (2000) 28:E63. 10.1093/nar/28.12.e63 10871386 PMC102748

[38] Avila HG Risso Marikena Guadalupe Ruybal P Repetto SA Butti MJ Trangoni MD . Development of a low cost copro-LAMP assay for simultaneous copro-detection of *Toxocara canis* and *Toxocara cati*. Parasitology. (2021) 17:1–34. 10.1017/S0031182021000342 33593468 PMC11010131

[39] Mabey D Peeling RW Ustianowski A Perkins MD. Diagnostics for the developing world. Nat Rev Microbiol. (2004) 2:231–40. 10.1038/nrmicro841 15083158

[40] Singh P Mirdha BR Ahuja V Singh S. Loop-mediated isothermal amplification (LAMP) assay for rapid detection of *Entamoeba histolytica* in amoebic liver abscess. World J Microbiol Biotechnol. (2013) 1:27–32. 10.1007/s11274-012-1154-7 23054695

[41] Han ET Watanabe R Sattabongkot J Khuntirat B Sirichaisinthop J Iriko H . Detection of four Plasmodium species by genus- and species-specific loop-mediated isothermal amplification for clinical diagnosis. J Clin Microbiol. (2007) 45:2521–8. 10.1128/JCM.02117-06 17567794 PMC1951264

[42] Karanis P Thekisoe O Kiouptsi K Ongerth J Igarashi I Inoue N. Development and preliminary evaluation of a loop-mediated isothermal amplification procedure for sensitive detection of Cryptosporidium oocysts in fecal and water samples. Appl Environ Microbiol. (2007) 73:5660–2. 10.1128/AEM.01152-07 17616628 PMC2042060

[43] Plutzer J Karanis P. Rapid identification of *Giardia duodenalis* by loop-mediated isothermal amplification (LAMP) from faecal and environmental samples and comparative findings by PCR and real-time PCR methods. Parasitol Res. (2009) 104:1527–33. 10.1007/s00436-009-1391-3 19288133

[44] De Ruiter CM Van Der Veer C Leeflang MMG Deborggraeve S Lucas C Adams ER. Molecular tools for diagnosis of visceral leishmaniasis: systematic review and meta-analysis of diagnostic test accuracy. J Clin Microbiol. (2014) 52:3147–55. 10.1128/JCM.00372-14 24829226 PMC4313130

[45] Karani M Sotiriadou I Plutzer J Karanis P. Bench-scale experiments for the development of a unified loop-mediated isothermal amplification (LAMP) assay for the *in vitro* diagnosis of Leishmania species' promastigotes. Epidemiol Infect. (2014) 142:1671–7. 10.1017/S0950268813002677 24168822 PMC9151212

[46] Gallas-Lindemann C Sotiriadou I Plutzer J Noack MJ Mahmoudi MR Karanis P. Giardia and Cryptosporidium spp. dissemination during wastewater treatment and comparative detection via immunofluorescence assay (IFA), nested polymerase chain reaction (nested PCR) and loop mediated isothermal amplification (LAMP). Acta Trop. (2016) 158:43–51. 10.1016/j.actatropica.2016.02.005 26880717

[47] Nzelu CO Kato H PN. Loop-mediated isothermal amplification (LAMP): an advanced molecular point-of-care technique for the detection of Leishmania infection. PLoS Negl Trop Dis. (2019) 13:e00076987. 10.1371/journal.pntd.0007698 31697673 PMC6837287

[48] Besuschio SA Llano Murcia M Benatar AF Monnerat S Cruz Mata I Picado de Puig A . Analytical sensitivity and specificity of a loop-mediated isothermal amplification (LAMP) kit prototype for detection of *Trypanosoma cruzi* DNA in human blood samples. PLoS Negl Trop Dis. (2017) 11:e0005779. 10.1371/journal.pntd.0005779 28727723 PMC5544240

[49] Avila HG Mozzoni C Trangoni MD Cravero SLP Pérez VM Valenzuela F . Development of a copro-LAMP assay for detection of several species of *Echinococcus granulosus* sensu lato complex. Vet Parasitol. (2020) 277:109017. 10.1016/j.vetpar.2019.109017 31901535

[50] Abdullah J Saffie N Sjasri FAR Husin A Abdul-Rahman Z Ismail A . Rapid detection of Salmonella Typhi by loop-mediated isothermal amplification (LAMP) method. Brazilian J Microbiol. (2014) 45:1385–91. 10.1590/S1517-83822014000400032 25763045 PMC4323314

[51] Pham NTK Trinh QD Khamrin P Ukarapol N Kongsricharoern T Yamazaki W . Loop-mediated isothermal amplification (LAMP) for detection of Campylobacter jejuni and *C. coli* in Thai children with diarrhea. Jpn J Infect Dis. (2015) 68:432–3. 10.7883/yoken.JJID.2014.450 25866115

[52] Bakhtiari S Alvandi A Pajavand H Navabi J Najafi F Abiri R. Development and diagnostic evaluation of loop-mediated isothermal amplification using a new gene target for rapid detection of helicobacter pylori. Jundishapur J Microbiol. (2016) 9:e28831. 10.5812/jjm.28831 27540449 PMC4976074

[53] Wang Y Wang Y Ma A Li D Luo L Liu D . The novel multiple inner primers-loop-mediated isothermal amplification (MIP-LAMP) for rapid detection and differentiation of listeria monocytogenes. Molecules. (2015) 20:21515–31. 10.3390/molecules201219787 26633345 PMC6332088

[54] Azizi M Zaferani M Cheong SH Abbaspourrad A. Pathogenic bacteria detection using RNA-based loop-mediated isothermal-amplification-assisted nucleic acid amplification via droplet microfluidics. ACS Sensors. (2019) 4:841–8. 10.1021/acssensors.8b01206 30908029

[55] Wang S Zheng L Cai G Liu N Liao M Li Y . A microfluidic biosensor for online and sensitive detection of *Salmonella typhimurium* using fluorescence labeling and smartphone video processing. Biosens Bioelectron. (2019) 1:111333. 10.1016/j.bios.2019.111333 31153017

[56] Löffler SG Leiva C Scialfa E Redondo L Florin-christensen M Martínez M . Detection of pathogenic leptospiral DNA traces in canine sera serum samples by loop mediated isothermal amplification (LAMP). Immunol Infect Dis. (2016) 4:39–43. 10.13189/iid.2016.040401

[57] Trangoni MD Gioffré AK Cerón Cucchi ME Caimi KC Ruybal P Zumárraga MJ . LAMP technology: rapid identification of Brucella and *Mycobacterium avium* subsp. paratuberculosis. Brazilian J Microbiol. (2015) 46:619–26. 10.1590/S1517-838246220131206 26273282 PMC4507559

[58] Telemann W. Eine methode zur erleichterung der auffindung von Parasiteneiern in den faeces. Dtsch Medizinische Wochenschrift. (1908) 35:1510–11. 10.1055/s-0028-1135692

[59] Repetto SA Soto CDA Cazorla SI Tayeldin ML Cuello S Lasala MB . An improved DNA isolation technique for PCR detection of Strongyloides stercoralis in stool samples. Acta Trop. (2013) 126:110–4. 10.1016/j.actatropica.2013.02.003 23416126

[60] Sambrook J Russell DW. Molecular Cloning: A Laboratory Manual. 3rd ed. New York, NY: Cold Spring Harbor Laboratory Press (2001). p. 10–4.

[61] Eiken. PrimerExplorerV5. (2018). Available online at: https://primerexplorer.jp/e/v5_manual/index.html (accessed June 4, 2020).

[62] Avila HG. Diagnóstico y Epidemiología Molecular de la Hidatidosis en terreno. Buenos Aires: EAE (2020).

[63] Jia WZ Yan HB Guo AJ Zhu XQ Wang YC Shi WG . Complete mitochondrial genomes of *Taenia multiceps, T. hydatigena* and *T. pisiformis*: additional molecular markers for a tapeworm genus of human and animal health significance. BMC Genomics. (2010) 11:447. 10.1186/1471-2164-11-447 20649981 PMC3091644

[64] Loukas A Hotez PJ Diemert D Yazdanbakhsh M McCarthy JS Correa-Oliveira R . Hookworm infection. Nat Rev Dis Prim. (2016) 2:16088 10.1038/nrdp.2016.88 27929101

[65] Hasslinger MA. Helminths of carnivores relevant to veterinary practice. Tierarztl Prax. (1986) 14:265–273. 3526635

[66] Traub RJ Robertson ID Irwin P Mencke N Thompson RCA. Application of a species-specific PCR-RFLP to identify Ancylostoma eggs directly from canine faeces. Vet Parasitol. (2004) 123:245–55. 10.1016/j.vetpar.2004.05.026 15325050

[67] Massetti L Colella V Zendejas PA Ng-Nguyen D Harriott L Marwedel L . High-throughput multiplex qPCRs for the surveillance of zoonotic species of canine hookworms. PLoS Negl Trop Dis. (2020) 14:e0008392. 10.1371/journal.pntd.0008392 32542036 PMC7316352

[68] Wong Y-P Othman S Lau Y-L Son R Chee H-Y. Loop mediated isothermal amplification (LAMP): a versatile technique for detection of microorganisms. J Appl Microbiol. (2017) 124:626–43. 10.1111/jam.13647 29165905 PMC7167136

[69] Njiru ZK. Loop-mediated isothermal amplification technology: towards point of care diagnostics. PLoS Negl Trop Dis. (2012) 6:1–4. 10.1371/journal.pntd.0001572 22745836 PMC3383729

[70] Ni XW McManus DP Lou ZZ Yang JF Yan H Bin Li L . A comparison of loop-mediated isothermal amplification (LAMP) with other surveillance tools for *Echinococcus granulosus* diagnosis in canine definitive hosts. PLoS ONE. (2014) 9:e100877. 10.1371/journal.pone.0100877 25007051 PMC4089910

[71] Bucher BJ Muchaamba G Kamber T Kronenberg PA Abdykerimov KK Isaev M . Lamp assay for the detection of echinococcus multilocularis eggs isolated from canine faeces by a cost-effective naoh-based dna extraction method. Pathogens. (2021) 10:847. 10.3390/pathogens10070847 34357996 PMC8308659

[72] Mugambi RM Agola EL Mwangi IN Kinyua J Shiraho EA Mkoji GM. Development and evaluation of a loop mediated isothermal amplification (LAMP) technique for the detection of hookworm (Necator americanus) infection in fecal samples. Paras Vec. (2015) 8:1–7. 10.1186/s13071-015-1183-9 26546069 PMC4636844

[73] Watts MR Kim R Ahuja V Robertson GJ Sultana Y Wehrhahn MC . Comparison of loop-mediated isothermal amplification and real-time PCR assays for detection of strongyloides larvae in different specimen matrices. J Clin Microbiol. (2019) 57:e01173–18. 10.1128/JCM.01173-18 30728195 PMC6440779

[74] Ngari MG Mwangi IN Njoroge MP Kinyua J Osuna FA Kimeu BM . Development and evaluation of a loop-mediated isothermal amplification (LAMP) diagnostic test for detection of whipworm, *Trichuris trichiura*, in faecal samples. J Helminthol. (2020) 94:e142. 10.1017/S0022149X2000022X 32238209

[75] Diakou A Di Cesare A Morelli S Colombo M Halos L Simonato G . Endoparasites and vector-borne pathogens in dogs from greek islands: pathogen distribution and zoonotic implications. PLoS Negl Trop Dis. (2019) 13:e0007003. 10.1371/journal.pntd.0007003 31067231 PMC6527238

[76] Simonato G Cassini R Morelli S Di Cesare A La Torre F Marcer F . Contamination of Italian parks with canine helminth eggs and health risk perception of the public. Prev Vet Med. (2019) 172:104788. 10.1016/j.prevetmed.2019.104788 31627164

[77] Jimenez Castro PD Mansour A Charles S Hostetler J Settje T Kulke D . Efficacy evaluation of anthelmintic products against an infection with the canine hookworm (Ancylostoma caninum) isolate Worthy 4.1F3P in dogs. Int J Parasitol Drugs Drug Resist. (2020) 13:22–27. 10.1016/j.ijpddr.2020.04.003 32403053 PMC7214830

[78] Castro PDJ Howell SB Schaefer JJ Avramenko RW Gilleard JS Kaplan RM. Multiple drug resistance in the canine hookworm *Ancylostoma caninum*: an emerging threat? Paras Vec. (2019) 12:576. 10.1186/s13071-019-3828-6 31818311 PMC6902405

[79] Jimenez Castro PD Venkatesan A Redman E Chen R Malatesta A Huff H . Multiple drug resistance in hookworms infecting greyhound dogs in the USA. Int J Parasitol Drugs Drug Resist. (2021) 17:107–17. 10.1016/j.ijpddr.2021.08.005 34492564 PMC8426179

