# Supplementary material for: Development of a New LAMP Assay for the Detection of Ancylostoma caninum DNA (Copro-LAMPAc) in Dog Fecal Samples
Source: Front Vet Sci. 2021 Nov 12;8:770508. doi: 10.3389/fvets.2021.770508 (PMC8633310; doi:10.3389/fvets.2021.770508)
Supplement: Supplementary file 1 [file Table_1.DOCX]

# Supplementary table 1

Copro-LAMPAc optimization of temperature and incubation time (negative control / 1pg *Ancylostoma caninum* genomic DNA).

|  |  | Time (min) | | | | | | | |  |
| --- | --- | --- | --- | --- | --- | --- | --- | --- | --- | --- |
|  |  | 15 | 30 | 45 | 60 | 75 | 90 | 105 | 120 |  |
| Temperature (ºC) | 52 | -/- | -/- | -/- | -/- | -/- | -/- | -/- | +/+ |  |
|  | 54 | -/- | -/- | -/- | -/- | -/- | -/- | -/- | +/+ |  |
|  | 56 | -/- | -/- | -/- | -/- | -/- | -/- | +/+ | +/+ |  |
|  | 58 | -/- | -/- | -/- | -/- | -/- | +/+ | +/+ | +/+ |  |
|  | 60 | -/- | -/- | -/- | -/+ | -/- | -/- | +/+ | +/+ |  |
|  | 62 | -/- | -/- | -/- | -/- | -/- | -/- | -/- | +/+ |  |
|  |  |  |  |  |  |  |  |  |  |  |
|  |  |  |  |  |  |  |  |  |  |  |
|  |  |  |  |  |  |  |  |  |  |  |
